# Repurposing existing skeletal spatial structure (SkS) system designs using the Field Information Modeling (FIM) framework for generative decision-support in future construction projects

**DOI:** 10.1038/s41598-023-46523-z

**Published:** 2023-11-10

**Authors:** Reza Maalek, Shahrokh Maalek

**Affiliations:** 1 Endowed Chair of Digital Engineering and Construction, Karlsruhe Institute of Technology, 76131 Karlsruhe, Germany; 2 Digital Innovation in Construction Engineering (DICE) Technologies, Calgary, T2N 0B3 Canada

**Keywords:** Civil engineering, Computer science

## Abstract

Skeletal spatial structure (SkS) systems are modular systems which have shown promise to support mass customization, and sustainability in construction. SkS have been used extensively in the reconstruction efforts since World War II, particularly to build geometrically flexible and free-form structures. By employing advanced digital engineering and construction practices, the existing SkS designs may be repurposed to generate new optimal designs that satisfy current construction demands of contemporary societies. To this end, this study investigated the application of point cloud processing using the Field Information Modeling (FIM) framework for the digital documentation and generative redesign of existing SkS systems. Three new algorithms were proposed to (i) expand FIM to include generative decision-support; (ii) generate as-built building information modeling (BIM) for SkS; and (iii) modularize SkS designs with repeating patterns for optimal production and supply chain management. These algorithms incorporated a host of new AI-inspired methods, including support vector machine (SVM) for decision support; Bayesian optimization for neighborhood definition; Bayesian Gaussian mixture clustering for modularization; and Monte Carlo stochastic multi-criteria decision making (MCDM) for selection of the top Pareto front solutions obtained by the non-dominant sorting Genetic Algorithm (NSGA II). The algorithms were tested and validated on four real-world point cloud datasets to solve two generative modeling problems, namely, engineering design optimization and facility location optimization. It was observed that the proposed Bayesian neighborhood definition outperformed particle swarm and uniform sampling by 34% and 27%, respectively. The proposed SVM-based linear feature detection outperformed k-means and spectral clustering by 56% and 9%, respectively. Finally, the NSGA II algorithm combined with the stochastic MCDM produced diverse “top four” solutions based on project-specific criteria. The results indicate promise for future utilization of the framework to produce training datasets for generative adversarial networks that generate new designs based only on stakeholder requirements.

## Introduction

### Skeletal spatial structure (SkS) systems in construction

**Figure 1:**
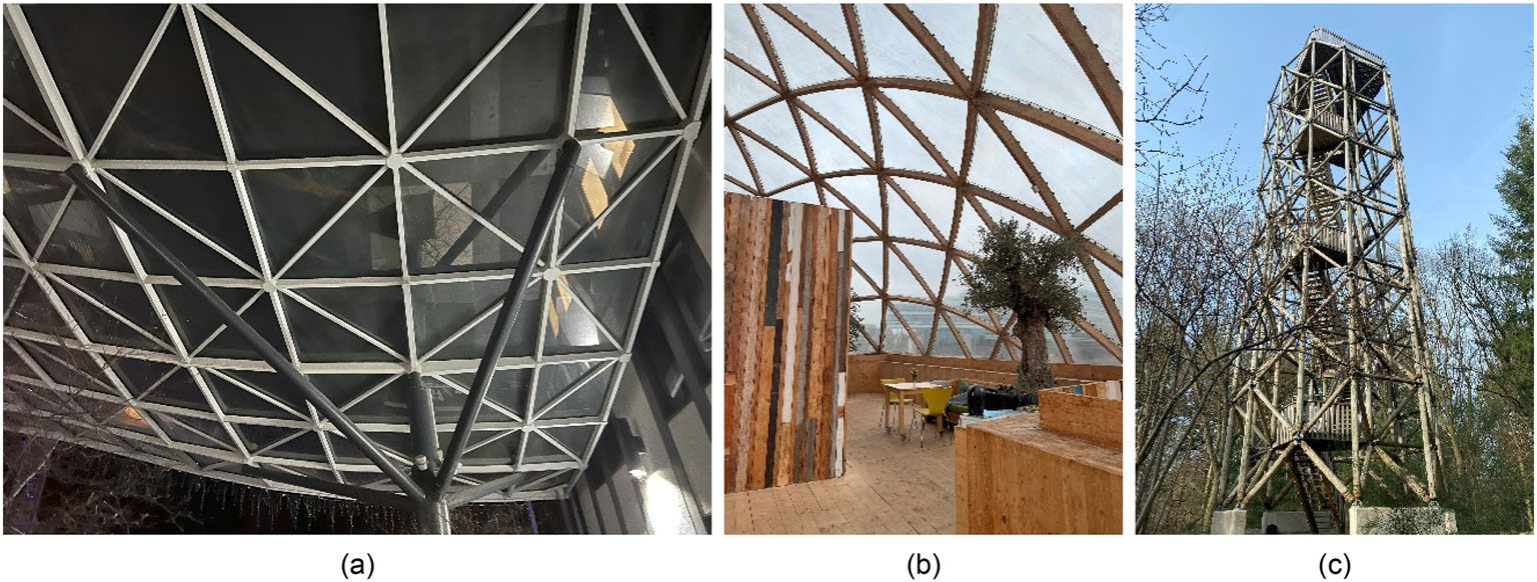
Examples of SkS: (**a**) free-form with linear members; (**b**) hemispherical with linear members; and (**c**) tower with cylindrical members.

Skeletal spatial structure (SkS) systems are modular structural systems, comprised of members arranged to create highly redundant 3-dimensional (3D) forms and joined together as truss and/or frame elements in single, double, or triple layered grids^1–3^. Figure 1 shows a few examples of SkS forms used in this study. Due to their high structural redundancy, the system becomes lightweight by design, directly reducing the embodied energy and embodied Carbon of the project, which are important aspects in construction sustainability^4^. Traditionally, the SkS systems together with industrial modularization practices, were adopted to support fast-paced and large-scale reconstruction after the second World War^2^. More recently, the SkS systems are utilized to design and construct architecturally aesthetic and geometrically free-form buildings and infrastructure. This latter property, when combined with lean industrial modularization practices in design and planning, enables the application of SkS systems for industrial mass customization^5^. Consequentially, advanced computational and digital engineering practices^6,7^ can be employed to design a single kit-of-parts solution that can be assembled in various configurations to generate versatile structural forms. Given the modular nature of SkS, the systems can be designed with disassembly, deconstruction, and recycling in mind, further supporting lifecycle sustainability by design.

The advantages of SkS systems compared to conventional systems include capacity for mass customization, reduced weight, increased structural redundancy, durability and stability, improved construction operations and management, and ease of dismantling, deconstruction, recycling, and reuse. The modular SkS systems, however, require an initial investment in training of personnel, along with the design and development of production facilities, which adhere to the best practices in lean manufacturing^8^ and total quality management (TQM)^9^, for optimal performance. This initial investment, hence, is subject to the principles of economies of scale^10,11^, proportional to the demands, to reach a desirable return on investment. As such, recent studies have focused on the application of SkS systems to address a large spectrum of sustainable construction demands in contemporary societies, such as bridges, including integral variable depth bridges^12^, residential buildings, offshore facilities, including wind turbines, and reinforcement/reconstruction of structures with cultural heritage significance^13^. These studies have shown promise for the large-scale application of SkS systems to substitute many conventional construction methods, particularly when collectively considering factors related to sustainability, stability, durability, and ease of construction, dissembling, reuse, and recycling.

### Utility of repurposing existing SkS designs

With the utilization and advent of locally sourced and new sustainable materials (e.g., engineered bamboo^14^), existing free-form SkS designs may be repurposed and optimized to achieve a fair balance between various construction performance aspects, including lifecycle sustainability, affordability, and quality. This new design may be utilized for new construction projects or to support sustainable reconstruction, renovation, and rehabilitation efforts of the existing structure when required. By virtue of its nature, repurposing existing designs as baseline to generate optimal designs in accordance with local requirements, exhibits several important advantages, some of which are:

- reduction of time and cost of engineering design (particularly schematic design), which constitutes 7.9–19% of total cost for new green construction projects, according to RSMeans -or on average 12.5% and 20–25% for traditional and building information modeling (BIM)-based^15^ projects, respectively, according to the Royal Architectural Institute of Canada^16^;
- reduction of uncertainty in intermediate and preliminary cost estimation of construction projects through increased maturity of the engineering design^17^;
- expediting the permitting process, which has been found to delay the start of construction by an average of 152.3 days in the developed countries of the Organisation for Economic Co-operation and Development (OECD), according to the World Bank^18^;
- providing baseline data and constraints for creating optimal designs, configurable to the requirements of a new project, particularly by utilizing generative modeling^19–21^, and artificial intelligence (AI)-based evolutionary and nature/biologically-inspired optimization processes^22–26^;
- enabling smaller architectural and consulting firms^27–29^-by employing effective digital transformation frameworks and competitive computational design practices- to design, make decisions (e.g., in procurement and supply chain^29^), and manage complex and larger projects, and consequently increase their competitive advantage in the market.

### Point clouds for digital documentation of SkS systems

The above benefits of repurposing the existing SkS system designs, however, can only be achieved with a large database of pre-existing designs, which may not be readily available, nor generally interoperable and of the same level-of-detail (LOD). For instance, only 2D drawings may be available in some instances and in others, 3D/4D BIM. In the case of BIM, while issue foundation class (IFC)^30^ provides standardization for improved interoperability, BIM with different LOD cannot simply be used together to train a machine learning model within current generative modeling frameworks. In this case, it is possible to standardize and regularize for consistency of all models through considerable manual intervention and revision. However, this is impractical, particularly for smaller firms with the goal of reducing the time and cost of engineering design through automation, and digitization. Therefore, this study provides an alternative approach to automatically generate semantic, and accurate as-built BIM of SkS systems using the Field Information Modeling (FIM)^®^^31^ process. By employing FIM^®^, various modes of data from the SkS systems, such as point clouds and images, may be utilized to automatically generate accurate, standardized, intelligent and semantic as-built BIM with the required (and consistent) LOD. The as-built BIM can then be employed for various purposes, such as training generative adversarial networks (GAN), and finding generative design optimization solutions, to solve a multitude of important engineering design and planning problems. More specifically, this study utilized FIM^®^ to automatically process point clouds acquired from four SkS systems in two separate classes. The generated model was then employed to define the boundary conditions and constraints in two case studies: (i) multi-objective generative optimization of engineering design to achieve a fair balance between structural performance, sustainability, and construction management; and (ii) optimal design modularization to support lean construction practices by design in the context of the generalized facility location problem (FLP).

### Manuscript structure

The remainder of the manuscript is composed of: (i) “Method” section, which includes the introduction to the state-of-the-art scientific gaps, followed by the proposed methods to address these gaps; (ii) “Experiments” section, which involves the explanation of the experimental design, benchmark methods used for comparison, and metrics for validation of the results; (iii) “Results” section presents the results of each of the scientific developments presented in the study; and (iv) “Discussion” section, which provides the summary of the scientific developments, results, and avenues for future research and developments. In particular, the “Method” section provides three new algorithms to provide: (i) the overall idea of FIM^®^ for point cloud processing in construction; (ii) the specific method to process point clouds of SkS systems using FIM^®^, including two AI-based scientific developments for Bayesian robust neighbourhood definition as well as SVM-based binary decision support; and (iii) the method to modularize SkS systems of typical double-layer grid structures into stable tetrahedral modules using a combination of frequent sub-graph mining and Gaussian mixture clustering.

## Method

### Filed Information Modeling (FIM)^®^ for generative decision-support in construction

**Figure 2:**
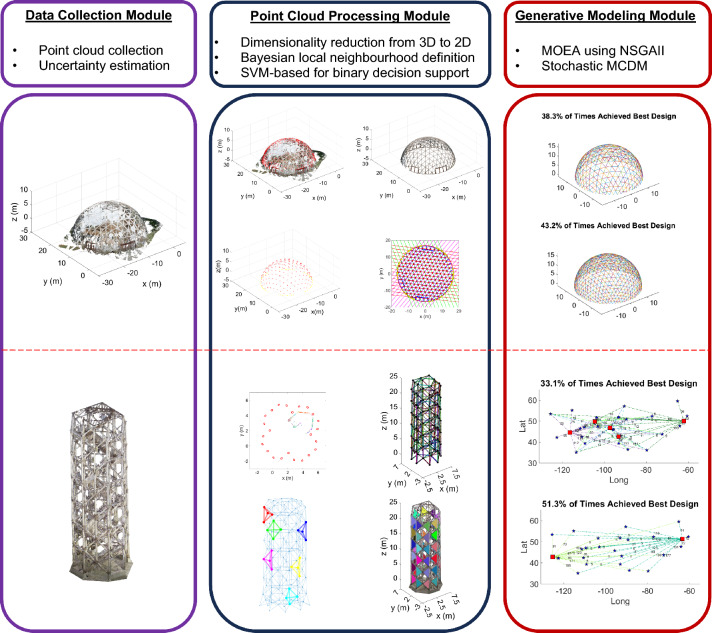
The process of integrating point cloud processing and generative modeling within the FIM framework for the two considered construction engineering and management problems: generative redesign of hemispherical SkS dome with linear members (top); and optimal modularization and facility location for SkS tower with cylindrical members (bottom).

FIM^®^^31^ is the process of transforming any field data (e.g., textual, audible, and visual) into intelligent, tangible, and semantic digital information as a means of enabling the seamless flow of information between the real and the digital worlds for effective situation assessment (e.g., SWOT analysis^32^) as well as data and decision-driven analytics^33^. Figure 2 provides the schematic representation of the three main modules of the proposed FIM^®^ framework for SkS member, namely, data collection module, point cloud processing module, and generative modeling module, to solve the two considered construction engineering and management problems, namely, the design optimization of hemispherical dome (Fig. 2-top), and the modularization and facility location optimization of a tower structure (Fig. 2-bottom).

### Filed Information Modeling (FIM)^®^ for point cloud processing in construction

In the context of visual information, 3D point clouds are commonly acquired using optical metrology, such as photogrammetry, and laser scanning, which must be automatically processed within the FIM^®^ process. Automated point cloud processing involves the automatic assignment of the acquired points to their corresponding real-world elements as defined in the n-D designed BIM, and/or technical specifications. Once the points are correctly assigned, they can be used to report progress^34^, detect dimensional incompatibilities^35^, update the design BIM^34^, generate digital twins^36^, or perform generative design optimization^19^. FIM^®^ for point cloud processing is advantageous since it incorporates all aspects of sensor characteristics (e.g., precision and calibration), construction errors, and a-priori baseline planned information into a single generic framework. This generic framework, inspired by^31^, is provided in Algorithm 1: point to BIM assignment below.

**Algorithm 1:** Point to BIM assignment.

**Inputs:** Project and design documentation, ${Pd}_{D}$; and Point cloud with source, ${Pt}_{C}$
**Outputs:** Assigned points to each design elements
1. Generate baseline information by classifying each functional element based on geometry, type, interdependencies, and schedule, along with the reference coordinate system, ${C}_{D}$, from ${Pd}_{D}$:
1.1. If BIM-based project, extract required information directly from the model;
1.2. Else-if CAD available, perform natural language processing on textual documents to link relevant information to CAD and generate BIM (go to step 1.a);
1.3. Else, perform image processing to generate 3D CAD from 2D drawings/blueprints (go to step 1.b)
2. Register point cloud, ${Pt}_{C}$ into the reference coordinate system, ${C}_{D}$
3. Assign points to their corresponding elements:
3.1. If construction error is absent, perform template matching between point cloud and BIM;
3.2. Else-if element model is represented analytically, perform robust model fitting of point cloud and BIM;
3.3. Else-if element model is geometrically composite and complex, perform local curvature analysis;
3.4. Else-if photogrammetric point clouds, perform steps 3.a-3.c by projective dimensionality reduction in 2D image plane

To formulate the details of the point cloud processing module in Fig. 2, it is important to provide the further information, particularly on the state-of-the-art and its gaps related to each step, presented in Algorithm 1.

#### Generation of baseline information

Gathering baseline information from a-priori and a-posteriori construction data involves the pre-processing of existing planned and design information, including textual information, 2D drawings and/or 3D/4D BIM. This process gathers strategically important information, such as material type, geometries, and proximity of objects that are expected to be present on the field at the time the point cloud data were collected. This baseline data is then used to determine the most appropriate strategy to process the point cloud. This stage can be further sub-categorized as follows:

- In the presence of BIM, the process commonly involves segmentation of elements in the BIM based on categories, such as functional type, geometry, and so on. This approach was employed for project controls using earned value management of reinforced concrete construction^34^.
- In the absence of a functional and intelligent BIM, natural language processing may be employed to gather the required information. This approach was utilized in^37^ to determine the planned radii and material of installed mechanical pipes.

#### Registration of point cloud into reference coordinate system

Registration of the point cloud to the coordinate system of the designed BIM (coarse registration in^38,39^). This can be accomplished through matching at least three non-collinear key-point correspondences between the plan and the point cloud. In the general case, these key-point correspondences must be taken at locations, independent from elements where possible construction errors are expected^35^.

#### State-of-the-art in point cloud processing

Here, the particular methodology in point cloud processing becomes dependent on the type of instrument for data collection, the expected level of construction errors (e.g., based on the chose construction materials and methods), and type of geometry and accuracy of available information on the element. In photogrammetric systems, the point cloud to element assignment is typically carried out by projecting the 3D BIM/CAD onto each image plane in the same pose^40,41^. The problem will be reduced to detecting element correspondences in 2D images, instead of 3D point clouds, which can capitalize on the many available and established image processing frameworks. The conversion of 3D BIM into 2D, however, is only suitable for images and cannot be trivially generalized to other instruments, such as terrestrial laser scanner (TLS). Furthermore, in the absence of a reliable BIM, supervised machine learning strategies, such as deep learning, must be utilized (e.g., brick walls^42^ and structural concrete^41^), which requires a large library of pre-classified objects, and consequentially presents extra hurdles for its widespread application.

Laser scanners (or active sensors), on the other hand, assign the 3D points to the baseline data by either template matching (in the absent of construction errors), robust model fitting (in the presence of analytical elements), local curvature analysis (in the presence of complex and composite element geometries), and machine learning (in the presence of experiential field point cloud data). Template matching includes the iterative closest point (ICP) template matching (scan vs. BIM and its variants)^38,43^, which decompose the model into points in the same density of the point cloud to perform iterative registration to match the point cloud and the model. In the presence of elements composed of many (more than three non-coparallel) planar surfaces, the point cloud vs. BIM method has shown to achieve better efficiency in terms of convergence rate and quality of final registration, compared to scan vs. BIM^31^. The template matching, however, can only be utilized in the presence of a 3D BIM with appropriate level of detail with the assumption of no construction errors^31^. The latter condition, however, cannot be generally guaranteed, particularly when construction quality control is the desired outcome^35,44^.

Robust model fitting to the point cloud^45^, which is appropriate when the geometry of the object of desire can be represented by some parametric equation (e.g., analytical shapes, such as planes^46–48^, and quadratics -cylinders^35,49^, spheres^50,51^ and ellipsoids^52^). Here, the problem is reduced to finding the points, amongst a collection of points, that follow the pattern of the desired parametric equation. This problem is typically handled through some robust Monte Carlo-based model fitting method, such as random sample and consensus (RANSAC)^53^, and least median of squares^54^ (or their many variants). Most robust model fitting methods, however, require a large set of initial sub-samples to guarantee an exact solution^35^. Therefore, approximate algorithms, which cannot generally guarantee the exact solution, must be utilized to preserve practicality^31^. Furthermore, when multiple of the same shape exists (e.g., multiple spheres to be detected), the model fitting must be performed repeatedly and sequentially (similar to recursive RANSAC^55^), until no additional shapes with the desired pattern can be found within the data, which in turn increases the computation time. In such cases, and cases with composite, and complex elements, which are composed of intersections of many surfaces and solids, it is advisable to first determine the potential groups of points that contain exactly one object with the desired pattern before performing the robust model fitting^35^. This can be achieved through local behaviour analysis as described below^31,56^.

Local behaviour analysis^31,56,57^, which is utilized to first select groups of points that locally follow a desired pattern. This approach is particularly beneficial when extraction of multiple analytical patterns (either of the same model or different models) from the same point cloud is desired. Local behaviour analysis starts by defining a local neighbourhood around each point. The pattern of the neighbourhood is then determined based on attributes, such as principal components analysis (PCA)^58^, Gaussian curvature^59,60^, and root mean squared errors^31^. The degree of agreement of the calculated attribute to that of a given class of surface geometry (e.g., planar, spherical, or cylindrical), which represent a proximate BIM element, is then determined. The incorporation of the local neighbourhood behaviour is attractive since it can limit the search space of the robust model fitting method by considering only points that locally follow the pattern of the considered surface geometry. The method, however, requires an efficient process to define the local neighbourhood around each point^31^. Therefore, adaptive variable neighbourhood sizes consistent with information theory^56^ and robust statistics^31^ were proposed. However, these methods rely on many uniformly distributed permutations of neighbourhood sizes, which may be impractical, and approximate. Other than the challenges associated with neighbourhood definition, the thresholds used to assign local points to a surface must also be flexible to point clouds acquired from different sources and scenes^31^. These thresholds are commonly defined subjectively in some of the existing literature (e.g., planes^46,47^ and cylinders^52,61^). In these cases, it may be beneficial to utilize prior data together with AI in particular machine learning strategies, to improve the subjectivity of the threshold definition.

Machine learning^62,63^, which can be utilized when historical relevant data exists (or can be artificially simulated) to perform complex decisions -in our case for semantic labeling without rigid and rule-based definition of subjective thresholds. Machine learning is advantageous to not only reduce dependencies on subjective and rigid thresholds, but also detect complex and non-analytical geometries (e.g., detection of boundaries of a cat from images). In the presence of historical data, supervised learning^34^ may be employed to perform tasks, such as detection of structural columns/beams and classification of reinforcement bars. Supervised learning generally requires a large library of pre-classified point clouds of common objects to populate the training dataset, which may neither be available nor practical. In the absence of historical data, unsupervised learning may be employed to perform point cloud clustering, such as k-means pipe clustering based on radii^37^, and hierarchical plane and line segmentation^58^. In other cases, semantic 4D BIM (i.e., with work sequencing information) together with active learning principles^64^ is shown to provide sufficient examples to define near misses^65^ and detect common construction elements, such as reinforced concrete columns, floors, and beams^58^.

#### Gaps in state-of-the-art in point cloud processing

**Figure 3:**
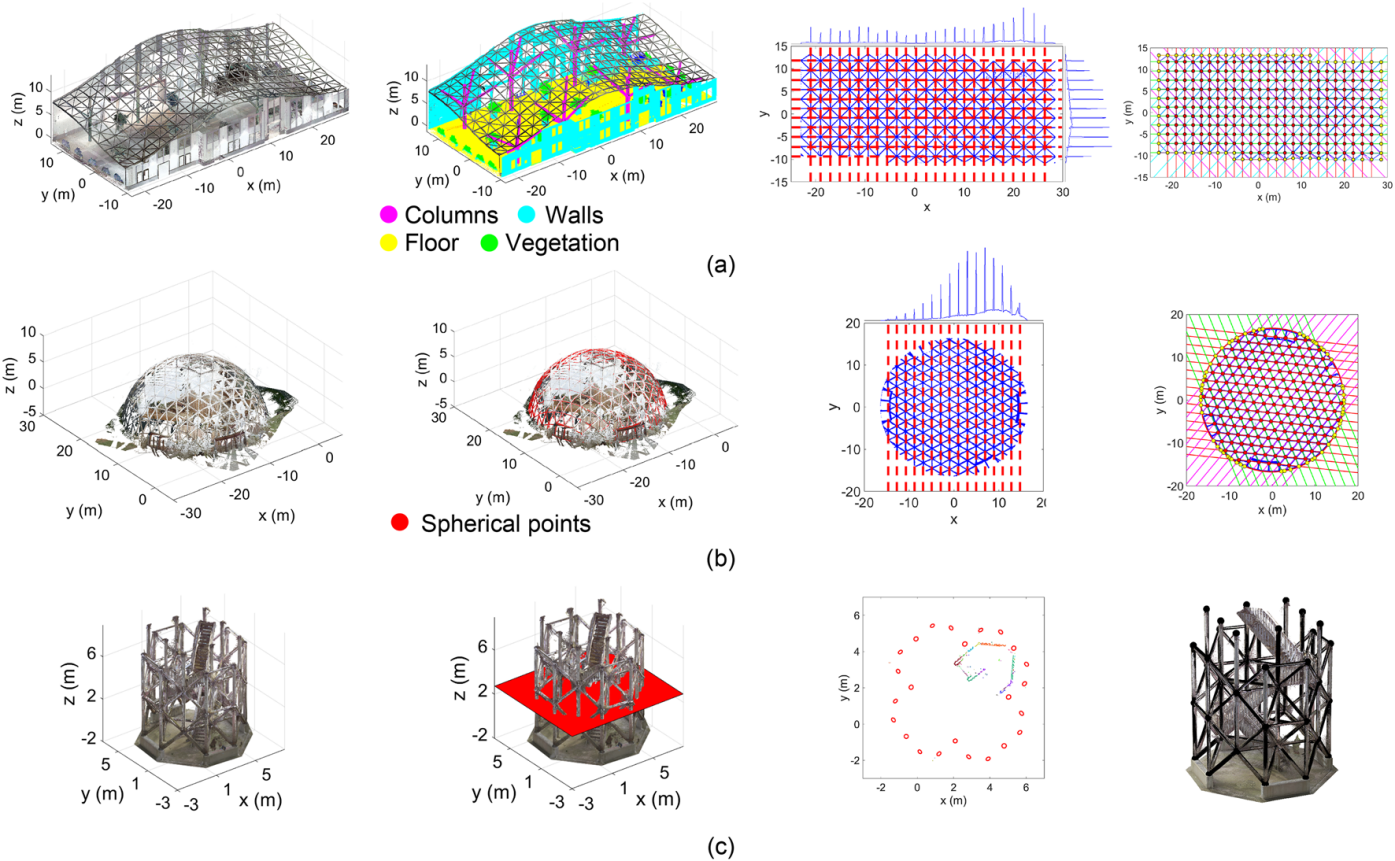
FIM for linear and cylindrical SkS -from left to right are steps 1 to 4 above: (**a**) linear free-form; (**b**) linear hemispherical (analytical arrangement); and (**c**) cylindrical.

While prior research provided solutions for automatic analysis of point clouds acquired from construction projects, the methods can become computationally expensive, the faster heuristic processing methods are typically biased to specific types of scenes or noise levels, and supervised learning methods require a large library of pre-classified point clouds^34,58,66^. In the following section, a new robust AI-based method for detection of two classes of SkS systems, namely, single-layer structures -free-form (Fig. 3a), and analytical (e.g., hemispherical; Fig. 3b) shells- with linear members, and structures with cylindrical members (Fig. 3c), is proposed.

### FIM^®^ for point cloud processing in SkS systems

With due consideration of the gaps in the state-of-the-art, a generic framework for automatic processing and semantic as-built BIM generation of SkS systems with two types of members, namely, linear, and cylindrical, is schematically provided in Fig. 3 and formulated in Algorithm 2: FIM^®^ for SkS point clouds as below.

**Algorithm 2:** FIM^®^ for SkS point clouds.

**Inputs:** Point cloud with source, ${Pt}_{C}$, and type of SkS member, here, linear free-form, ${L}_{F}$ (Fig. 3a), linear analytic, ${L}_{A}$ (Fig. 3b), and cylindrical ${C}_{D}$
**Outputs:** Analytic 3D structural BIM of SkS, including nodes, elements (e.g., size and connectivity), and location of supports
1. **SkS Region of Interest (ROI)** (Fig. 3-far-left)**:** Determine the ROI with primarily points of SkS members:
1.1. If SkS type is ${L}_{F}$ (Fig. 3a-far-left), remove points of common urban objects using the following:
1.1.1. Utilize the method of^58^ to remove planar surfaces, including floors, beams, slabs and walls;
1.1.2. Utilize the method of^67^ to remove columns and pipes;
1.1.3. Utilize the method of^68^ to remove vegetation
1.2. Else-if SkS type is ${L}_{A}$ (Fig. 3b-far-left), extraction of points of the a-priori analytical geometry using the robust AI-based heuristic model fitting of^31,50,51^
1.3. Else-if SkS type is ${C}_{D}$ (Fig. 3c-far-left) define the cut-planes for cylinder detection using the method of^66,67^
2. **Dimensionality Reduction** (Fig. 3-middle-left): Reduce the dimension of the ROI points from 3D into 2D:
2.1. If SkS type is ${L}_{F}$ or ${L}_{A}$ (Fig. 3a and b-middle-left), arrange the linear members into Euclidian tiling using projection;
2.2. Else-if SkS type is ${C}_{D}$ (Fig. 3c-middle-left), determine the proximate points to cut-planes using orthogonal projection
3. **Local Curvature Analysis** (Fig. 3-middle-right): Perform local behaviour analysis on 2D projected points:
3.1. If SkS type is ${L}_{F}$ or ${L}_{A}$ (Fig. 3a and b-middle-right), detect linear 2D features using the following:
3.1.1. **Robust Neighbourhood Definition:** Find the neighbourhood of points that minimizes the normalized covariance determinant (Algorithm 2 of^31^) using AI-based Bayesian optimization;
3.1.2. **Principal Component Analysis (PCA)**: Perform PCA on the robust neighbourhood of step 3.1.1;
3.1.3. **Peak Detection:** Detect the peaks of estimated angles of the directional vector using method of^35^;
3.1.4. **Axis Alignment:** For each detected peak, find the rotation that aligns the direction vector onto the y-axis;
3.1.5. **Dimensionality Reduction:** for each rotation, project rotated points onto the x-axis (1D data);
3.1.6. **SVM-based Binary Decision:** detect linear features by applying SVM on 1D data
3.2. Else-if SkS type is ${C}_{D}$ (Fig. 3c-middle-right), detect elliptical 2D features using the following:
3.2.1. **Robust Neighbourhood Definition:** Find the neighbourhood of points that minimizes the normalized covariance determinant (Algorithm 2 of^31^) using AI-based Bayesian optimization;
3.2.2. **Connected Components:** Find the connected points using region growing on closest neighbours;
3.2.3. **Ellipse Fitting:** Fit ellipses to each connected region using the method of^69^;
3.2.4. **Euclidian Ellipticity:** estimate the Euclidian ellipticity for each candidate elliptic region using^67^;
3.2.5. **SVM-based Binary Decision:** detect elliptic features by applying SVM on the Euclidian ellipticity
4. **Generate Structural BIM of SkS** (Fig. 3-far-right): Determine the node coordinate, element, and connectivity:
4.1. If SkS type is ${L}_{F}$ (Fig. 3a-far-right), generate structural BIM of linear free-form elements as follows:
4.1.1. **2D Node Coordinates**: Estimate candidate node coordinates by intersection of detected 2D gridlines;
4.1.2. **Element Segmentation**: Segment linear elements between two connected nodes in the direction of gridline;
4.1.3. **3D Line Fitting**: Fit robust 3D lines to each segmented element;
4.1.4. **3D Node Coordinates**: Intersect 3D lines of neighbouring elements
4.2. Else-if SkS type is ${L}_{A}$ (Fig. 3b-far-right), generate structural BIM of linear analytic SkS as follows:
4.2.1. **2D Node Coordinates**: Estimate candidate node coordinates by intersection of detected 2D gridlines;
4.2.2. **Element Segmentation**: Segment linear elements between two connected nodes in the direction of gridline;
4.2.3. **3D Node Coordinates**: Back-project 2D node coordinates onto the a-priori analytical geometry of SkS
4.3. Else-if SkS type is ${C}_{D}$ (Fig. 3c-far-right), generate structural BIM of cylindrical SkS elements as follows:
4.3.1. **Cylinder Parameter Estimation**: Estimate cylinder parameters from detected ellipses using the method of^67^;
4.3.2. **3D Node Coordinates**: Intersect the axis of neighbouring cylindrs using a bundle least squares adjustment;
4.3.3. **Cylinder Parameter Adjustment:** Adjust each element’s cylinder axis using the 3D node coordinates

In the following section, two important AI-based scientific developments related to Algorithm 2, namely, the Bayesian robust neighborhood definition (stages 3.1.1 and 3.2.1), and SVM binary decision support (stages 3.1.6 and 3.2.5), are explained in more detail.

### Bayesian robust neighborhood definition

Performing local behavior analysis (Algorithm 1-stage 3.3) requires robust and adaptive neighborhood definition. The traditional procedures introduced in^56^ and^31^ found the adaptive neighborhood by means of uniform sampling. The neighborhood size, achieving the minimum of the objective function (eigentropy^56^ and minimum covariance determinant (MCD)^31^), was selected as the optimal neighborhood size for each point. Uniform sampling, however, cannot guarantee the correct solution and may require more than necessary function evaluations, which reduce the efficiency of the method. Given that the closed-form solution that maps the neighborhood size to the objective functions cannot be formulated (i.e., the gradient of the object function cannot be estimated), AI-based optimization algorithms, inspired from natural and evolutionary processes, might provide an alternative option to guide the direction of movement of each iteration to achieve a reliable solution. To this end, the problem is formulated as a combinatorial optimization process and solved using the Bayesian optimization^70,71^ to find the optimal arrangement of neighbors around each point, more specifically, the set of points that achieve MCD. In the “Results” section, it will be empirically shown that the proposed optimization outperformed uniform sampling, and particle swarm algorithm in this context.

### SVM for binary decision support

In this study, a type of statistical supervised learning, called, SVM^72,73^, is utilized to aid with binary decisions (i.e., decisions of accept or reject). Given a set of training data, SVM provides the best hyperplane that separates the data, in our case, into binary classes. This hyperplane is then utilized to predict the binary class for a new dataset. Since SVM requires training data, here, a Monte-Carlo simulation approach is utilized to generate large training datasets for the SVM classifiers. In this study, three types of SVM classifiers are trained, to:

- provide a rule-of-thumb definition of linear vs. cylindrical elements as input for Algorithm 2;
- select prominent modes for linear feature extraction (stage 3.1.6); and
- differentiate between elliptic and non-elliptic points for cylindrical feature extraction (stage 3.2.5).

#### Linear vs. cylindrical SkS

**Figure 4:**
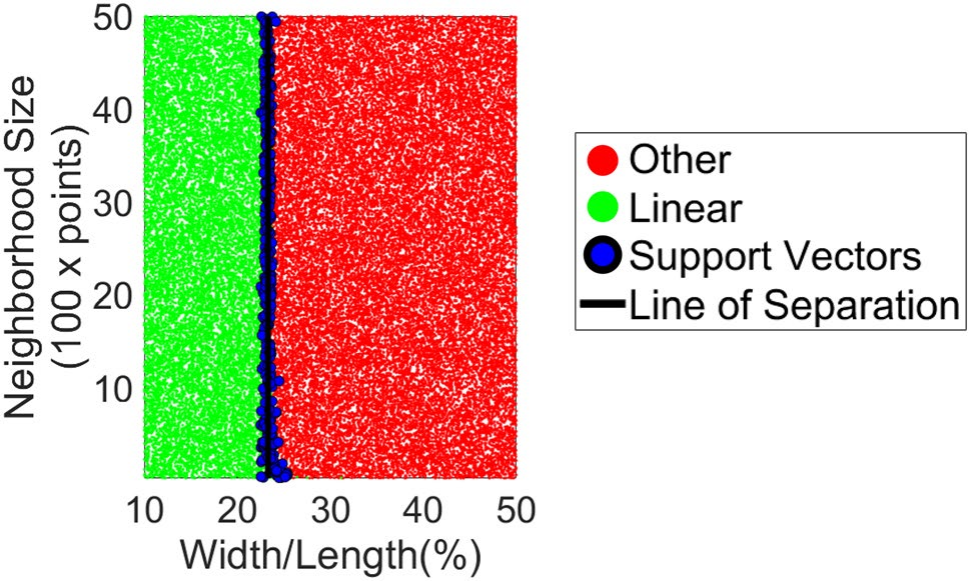
Support Vector Machine (SVM) classifier for linear vs. non-linear elements.

Given that a perfectly linear element in 3D does not exist in nature, following the convention set by^58^, a linear element is formally defined as an element with the largest surface variation ratio^74^ of 0.9 and 0.95 in 3D and 2D, respectively. Here, the goal is to provide a rule-of-thumb definition of linearity based on width/depth to length ratio of an element, instead of more complex eigenvalue-based definitions. To provide a rule of thumb definition of linearity, one million combinations of points in 2D and 3D were generated and classified based on the binary surface variation thresholds (i.e., 0.9 and 0.95, respectively)^75^. Each set of points varied based on number of points, width to length ratio, and measurement error. For each set, the surface variation ratio was then calculated and used to classify the surface as linear and non-linear based on the formal thresholds. SVM is then utilized to determine the separable ratio of width to length of the element in 2D and 3D. The results, provided in Fig. 4, demonstrate that for dense points with at least 100 neighboring points, an element is considered linear when the width to length ratio is 23.38% with a cross-validation loss of approximately 0.11%. This number is also consistent with the analytically derived square root of the ratio between the smallest and largest eigenvalues for the largest surface variation ratios of 0.9 and 0.95, which are 23.6% and 22.9%, respectively, for a symmetric element. The rule of thumb ratio, 23.38%, was used throughout the manuscript to differentiate between linear and cylindrical SkS elements from the plan model (i.e., as described in Algorithm 1 stage 1) as input for Algorithm 2.

#### Linear feature detection from prominent modes

**Figure 5:**
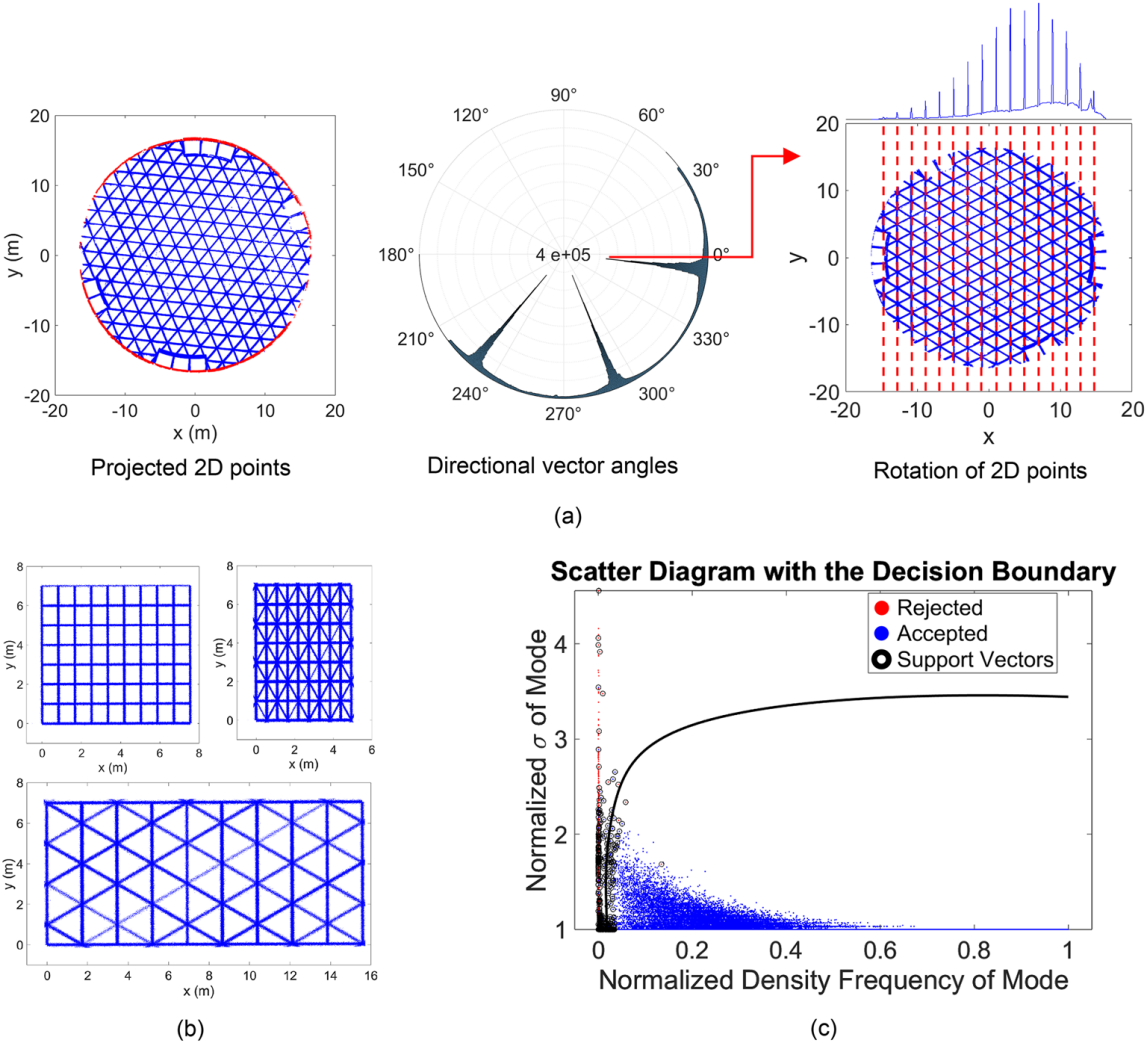
(**a**) General framework for line detection in 2D: projected 2D points with detected boundaries in red (left); polar histogram of the angle between the directional vector and x-axis (middle); detected grid lines of rotated points in red (right). (**b**) sample of Euclidian tiling patterns for the simulated training datasets; and (**c**) results of the SVM training of the simulated data.

Stage 3.1.5 of Algorithm 2 requires training of SVM to classify linear from non-linear features using information gathered during robust PCA (stages 3.1.1 and 3.1.2). Given the regular nature of modular SkS in 2D projections (see Fig. 5a and b), the process of line detection in 2D is first reduced to mode detection in 1D through a rotation. Inspired by the floor detection in^34^, the modes of the directional vectors corresponding to the largest eigenvalues of the principal component is used as bases to rotate the points such that the directional vector becomes parallel to the y-axis (Fig. 5a). The problem is then reduced to detecting the prominent modes of 1D rotated data in the x-axis direction. Other than the improved computation through dimensionality reduction from 2D to 1D, this separation of rotation and intercept also circumvents the inconsistent and variable scaling between the directional vectors and the intercept, commonly observed in Hough Transform-based line detection methods. Here, the main challenge is to effectively separate the prominent modes of the x-axis components of the rotated points from other less significant peaks. To this end, a collection of 10 million sets of patterns, inspired by common modular SkS systems were generated. Amongst these arrangements, geodesic, and Euclidean tilling, such as Tetrakis square, triangular (deltille), and regular square (Fig. 5b), patterns were generated. For each simulated element of the dataset, the maximum width to length ratio of 23.38% (Fig. 4) was respected. Each dataset differed in terms of pattern, number of points, local point density, rotation error (for directional vector alignment to the y-axis) and noise. To train the data, the actual modes from the simulated points were first generated and used to classify correct from incorrect peaks. These peaks were detected using the mode detection process described in^35^. Two variables, namely width and frequency of the estimated peaks were adopted to train the SVM. Figure 5c shows the results of the trained SVM on all datasets with a cross-validation loss of approximately 0.09%. The results indicated that modes with properties on the right of the curve (blue points) are considered prominent and represent a linear feature. As will be empirically observed in the results, the proposed methodology for line detection with SVM peak detection considerably outperformed binary clustering using unsupervised learning methods such as k-means and spectral clustering.

#### Elliptic feature detection from connected points

**Figure 6:**
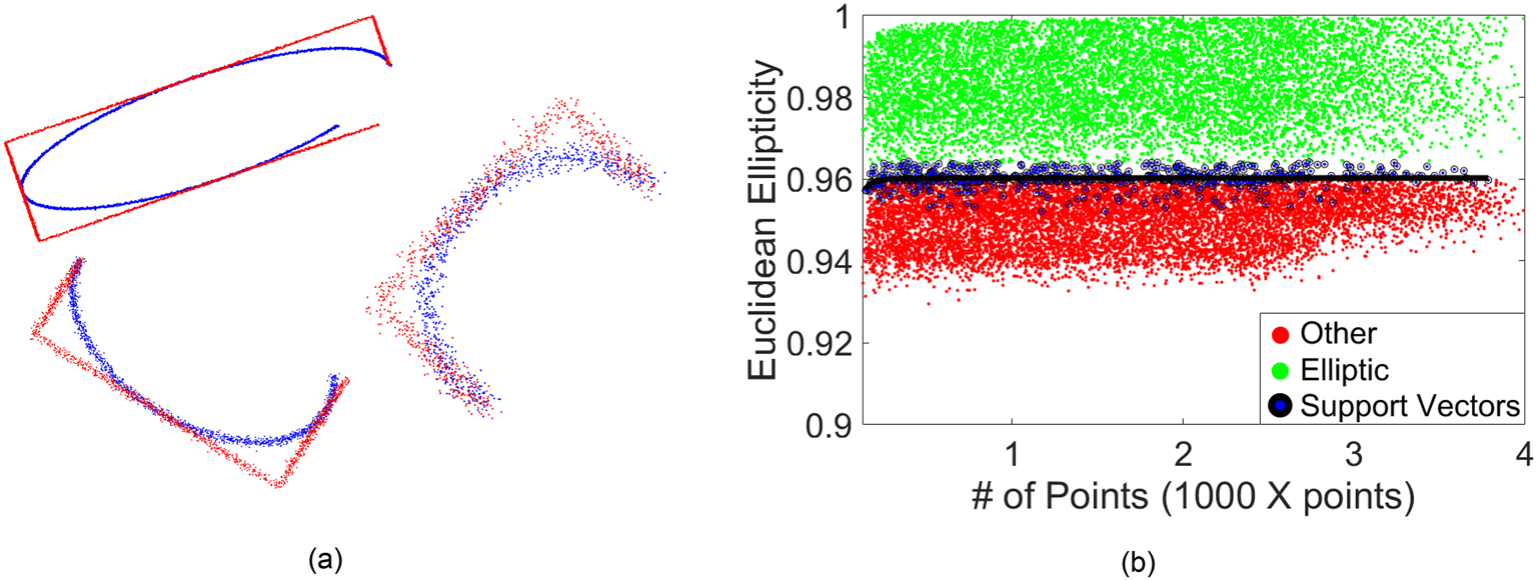
(**a**) Samples of simulated points following elliptic (blue) and rectangular/other (red) patterns; (**b**) results of the SVM and curve of separation between elliptic and non-elliptic shapes.

In^67^, an approach to separate elliptic from non-elliptic points in digital images was proposed, which used Monte-Carlo simulation together with the Euclidean ellipticity metric. Here, that method was extended to projected 2D point clouds (Fig. 6a) through the utilization of supervised learning best practices, i.e., SVM. The results of the SVM training is provided in Fig. 6b. The results indicated that the Euclidean ellipticity asymptotically yields to 0.96 for number of points larger than 200. This finding agrees with that presented in Figures 13, 14 and 15 of^67^.

### Generative modeling and optimization

The previous section provided the practical approach for semantic as-built BIM generation of SkS members using FIM^®^. This as-built BIM can be utilized as baseline design to generate optimal designs that achieve a fair balance between multiple criteria, such as sustainability, accessibility, affordability, and efficiency. Solutions to these complex construction engineering and management problems require the simultaneous satisfaction of multiple important, but conflicting objectives and conditions. Furthermore, in many instances, the relationship between the decision variables and objective functions cannot be represented in closed-form. As such, classical deterministic optimization approaches, such as Gauss–Newton and Levenberg–Marquardt, connot be utilized since the gradient of the objective functions (i.e., the direction of movement from an initial estimate) cannot be formulated. In such cases, AI-based optimization methods can be employed to generate Pareto efficient solutions (the Pareto front). In this study, the non-dominated sorting Genetic Algorithm (NSGA-II)^22,23,76^ is employed, which has shown to provide efficient solutions to complex multi-objective construction engineering and management optimization problems^77^. For this, two case studies from real-world SkS projects are utilized as baseline for the generative optimization. The first is the design optimization of the hemispherical dome of Fig. 3b to achieve a fair balance between embodied energy, embodied Carbon, weight, volume, strain energy, fundamental frequency, and construction cost. The second is the facility location optimization problem for modularization of the elements of the tower shown in Fig. 3c to achieve a fair balance between Carbon footprint, regularity of the capacity distribution, and cost.

#### Stochastic multi-criteria decision-making (MCDM) on Pareto front solutions

The NSGA II algorithm provides many Pareto front solutions. At this stage, the project team must select a few design alternatives from the many generated designs that best satisfy the requirements of the project. This problem can be likened to the MCDM problem of selecting an alternative (e.g., selection of a car from a set of available cars) based on factors/criteria (e.g., cost, quality, fuel efficiency), representing the design alternatives, and the objective functions in the present problem, respectively. A host of methods, such as analytic hierarchy process (AHP)^78^, and best–worst method^79^, have been proposed in the literature to support with MCDM. In the case of the present problem of generative design, the value of the objective functions for each design alternative is already generated by the NSGA II algorithm. As such, an effective strategy must be deployed to weigh the relative importance of the objective functions. In AHP and best–worst methods, this relative weighting is performed manually. While it is possible to hold a stakeholder engagement session (or through distribution of online surveys) to complete the weighting, the individual weights may become biased, grossly different, and subjective in real-world projects. In practice, the authors have found that instead of requesting concrete numbers as relative importance, it is much more flexible and practical to build consensus with the project stakeholders on the order of the relative importance of the objectives. Once the order of importance is determined collectively, a Monte-Carlo approach is utilized to stochastically determine the repeating solutions by assigning many random values as the importance weight between different objectives. Using this Fuzzy weighting approach, the percentage of times a particular design alternative achieved the best score was quantified. In the spirit of the magic number 4 in human cognitive capacity^80^, the top four solutions are reported and utilized by the project team to select the final design. At this stage, the design team can efficiently and objectively decide on the final solution from only the four final solutions, instead of all generated Pareto front solutions, reducing the possibility of decision fatigue^81^.

#### Hemispherical dome design optimization

**Figure 7:**
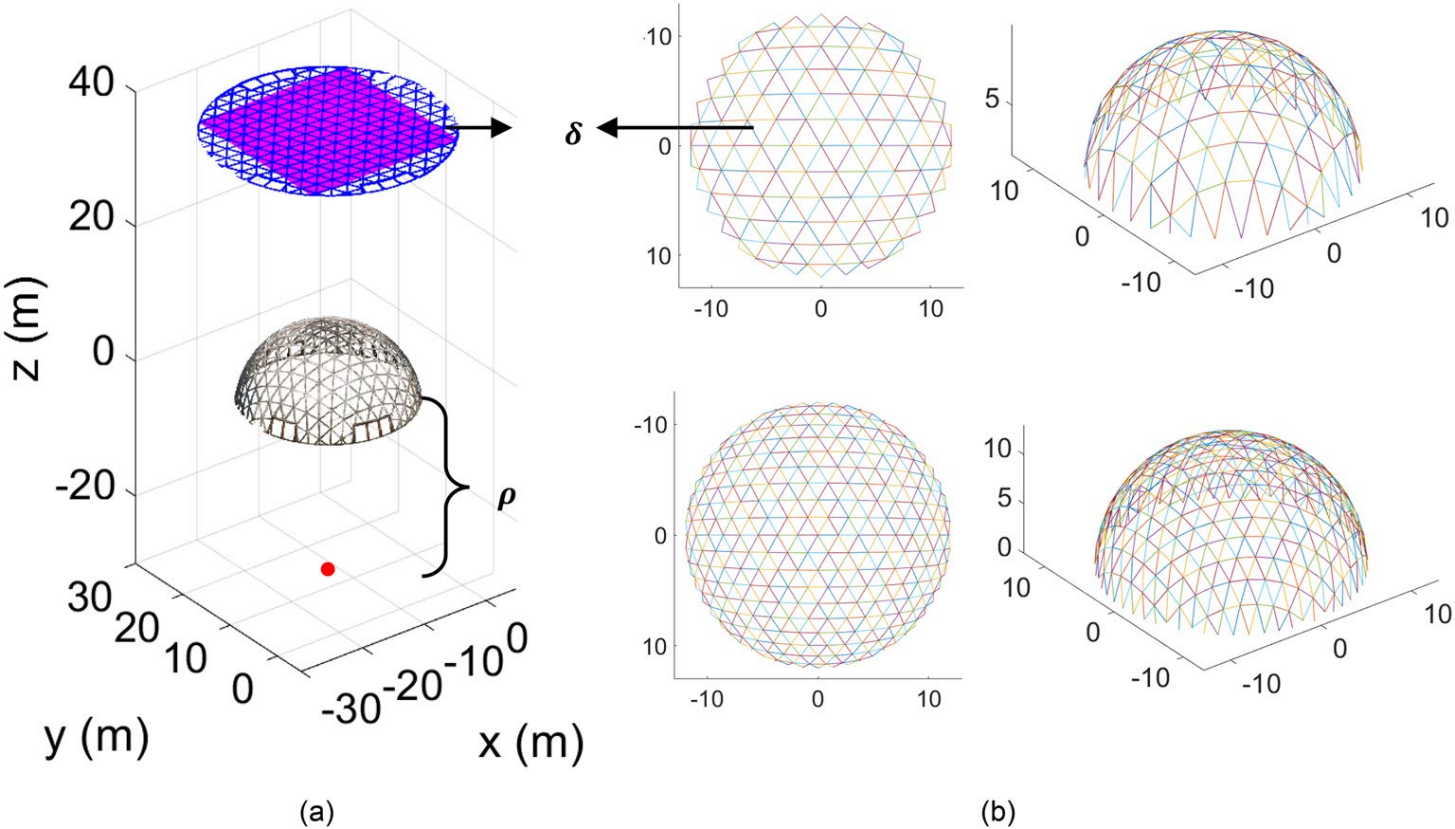
Generative dome design optimization decision variables: (**a**) parametric construction of the dome nodes and elements from perspective projection; (**b**) two examples of design alternatives by changing two of the decision variables.

Generative optimization requires a set of decision variables and multiple objectives that can be numerically estimated given the value (or type) of the decision variables. Based on the original design documents, the hemispherical dome considered in this study was generated through a perspective back-projection of a planar triangular uniform Euclidean tiling (deltille) onto a hemisphere. Figure 7a demonstrates this projective transformation. In the original design (Fig. 3b), the perspective center was set at one diameter below the center of the hemisphere. Here, to generate different designs from the original design, three decision variables are selected, namely, the location of the perspective center ($\rho$), the size of the edge of the deltille ($\delta$), and three material types, namely, steel, aluminum, and wood. Figure 7b shows two sample new designs with different $\rho$ and $\delta$ in 2D (left) and 3D (right).

For each design alternative, a set of objective functions were formulated. To this end, the following steps were performed:

- Optimal weight of structure, which is achieved through structural topology optimization best practices^82^. For this, first, an initial area for each element is assumed. Relevant static and dynamic loads (dead, live, wind, and snow) are defined based on established standards, here, the national building code (NBC) of Canada^83^. Finite element analysis (FEM) is then performed to estimate the relevant structural responses, such as member’s stress and displacement. The area of each member is then iteratively imporoved such that the load and resistance factor design (LRFD) standards for steel^84^, aluminum^85^, and wood^86^ elements are satisfied. The process is iterated until the weight between two consecutive iterations remain the same. The final weight is then used to calculate the following items.
- Volume is calculated by means of the optimal weight divided by the material density.
- Embodied energy and Carbon are calculated by multiplying the Inventory of Carbon and Energy (ICE) unit values by the optimal weight.
- Strain energy is calculated proportional to the stiffness multiplied by squared displacement, and fundamental frequency is calculated as the square root of the smallest generalized eigenvalue of the stiffness and mass matrices.
- Construction cost is calculated using RSMeans unit pricing and the quantity of material (weight/volume), along with a factor proportional to square root of the number of nodes (to account for economy of scale and assembly learning curve) of the considered design.

The fundamental frequency must be maximized, while weight, embodied energy, embodied Carbon, volume, strain energy and cost must be minimized.

#### Spatial tower modularization for assembling facility location optimization

**Figure 8:**
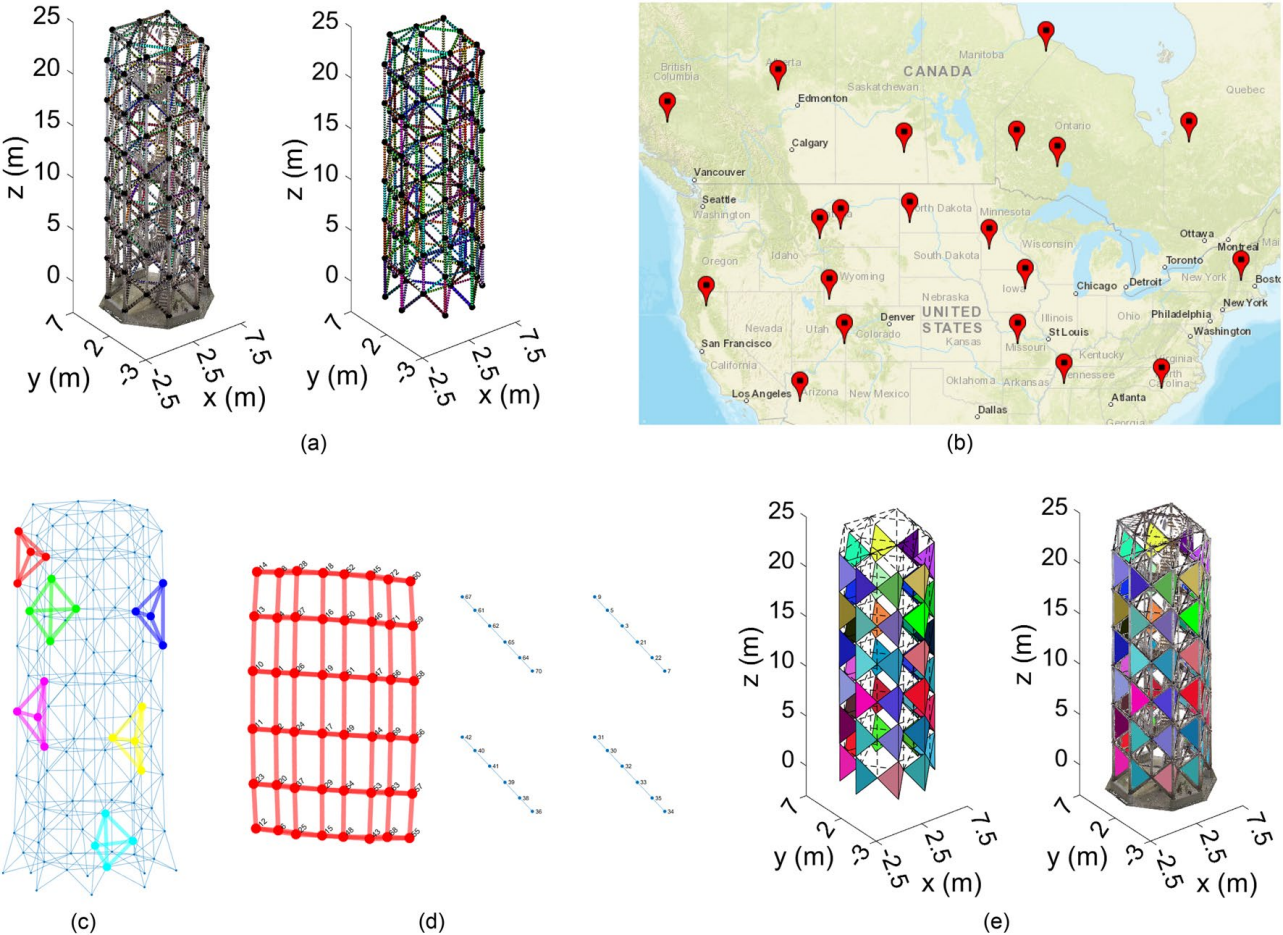
Automated tetrahedral modularization of SkS tower: (**a**) detected cylindrical elements and nodes- output of Algorithm 2; (**b**) locations of site for new towers (generated using Matlab Web Map^91^ and Web Marker^92^); (**c**) graph representation of the spatial truss along with six sample detected cliques of size four (tetrahedral modules); (**d**) detection of longest connected modules; and (**e**) final detected modules- output of Algorithm 3.

Let’s assume that the project team is interested in rebuilding different sizes and heights of spatial towers of the type, shown in Fig. 8a, across North America (Fig. 8b). The goal is to: (i) modularize the existing tower from the outputs of Algorithm 2; and (ii) build temporary facilities for assembly of the modules and its distribution to the sites based on their demand. The latter can be optimized using the NSGA II algorithm as a FLP. The original FLP, however, is limited to the selection of $k$ out of $n$ facilities ($n\ge k$) with known locations, which is by itself NP-hard combinatorial optimization problem. In this study, no constraint is provided for the locations of facilities to provide more flexibility, albeit more challenging. The optimization, here, considers three factors, namely, Carbon footprint of transportation, uniformity of the facility capacities, and cost of facility. Given the decision variables of location and number of facilities, along with the input demand of each site, the following steps were performed:

- Capacity optimization, which includes solving a mixed integer linear program^87^ to determine the capacity of each facility and the number of modules supplied to each site. Here, the cost of supply of a module from a facility to a site is assumed proportional to the square of the distance between the facility and site.
- Uniformity of facility capacity, which is defined as the standard deviation of the optimal capacity of the facilities.
- Carbon footprint, which is calculated proportional to the sum of the distance of each facility to the site multiplied by the corresponding optimal capacity.
- Cost of facility, which is calculated based on capacity proportions followed by unit pricing (adjusted for facility size, and location) for construction of temporary facilities adopted from RSMeans data.

The generative optimization must find the Pareto front solutions for the number and locations of facilities that minimize uniformity of capacity, Carbon footprint, and cost.

#### Modularization of spatial tower

In this section, an automated process for identifying repeatable modules in SkS systems, specifically those with repeating polyhedral patterns, is proposed. For spatial trusses (SkS members with hinge connections), members forming tetrahedral shapes are statically stable in 3D, and are commonly utilized as repeatable modules to generate double- or triple-layered grid decks. Similarly, a new method is proposed, here, to divide the SkS tower into tetrahedral modules. To this end, the SkS tower is treated as a graph with edges and vertices as the cylindrical members and the nodes, respectively. Once converted into a graph, the problem of detecting tetrahedral modules from BIM transforms to detecting cliques (complete subgraphs) of size 4 (4 vertices). The most frequent connected modules where every two module shares at most one node (no edges) are then determined. All edges of the detected modules are removed, and the process is continued until no additional modules can be found. This process is explained in Algorithm 3: Tetrahedral modularization of SkS, below.

**Algorithm 3:** Tetrahedral modularization of SkS.

**Inputs:** Generated Structural SkS BIM, ${SkS}_{BIM}$
**Outputs:** Modules of stable tetrahedral arrangement
1. **Graph Transformation of** ${{\varvec{S}}{\varvec{k}}{\varvec{S}}}_{{\varvec{B}}{\varvec{I}}{\varvec{M}}}$ (Fig. 8c)**:** Transform ${SkS}_{BIM}$ into undirected graph network with connection and cylinderical members as node and edges, respectively
2. **Tetrahedral Modularization:** Find all frequent cliques of size four (modules) recursively as follows:
2.1. **Cliques of Size Four** (Fig. 8c)**:** find all modules in the undirected graph using the Bron-Kerbosch method^88,89^;
2.2. **Frequent Clusters of Connected Cliques** (Fig. 8d)**:** Find the frequent clusters of connected modules as follows:
2.2.1. For each module, determine all other modules that share exactly one node;
2.2.2. Generate a new graph by utilizing the module connectivity and adjacency matrix from step 2.2.1;
2.2.3. Determine connected subgraphs of the generated graph in step 2.2.2 using connected components;
2.2.4. Calculate the length of each connected set of modules;
2.2.5. Select the longest connected set of modules;
2.2.6. Remove all edges associated with the longest connected modules from the remaining modules;
2.2.7. Repeat steps 1–4 above until no additional modules is remained
2.3. **Module Clustering** (Fig. 8e)**:** Cluster modules with similar edge lengths from all identified modules from steps 2.2
2.3.1. For each module, generate the matrix of edge lengths;
2.3.2. For each module, estimate the edge length standard deviations using the law of propagation of error on the estimated node coordinate covariances from Algorithm 2-stage 4.3.3;
2.3.3. Estimate the Mahalanobis distance of the lengths between every two module;
2.3.4. Generate new adjacency matrix for the modules, where two modules are considered adjacent (i.e., array value of is “1”) if the Mahalanobis distance between the module lengths is less than $\sqrt{{\chi }_{\mathrm{0.95,6}}^{2}}=3.5485$ -the square root of the Chi squared probability with degree 6 (for the six edges) and 95% confidence
2.3.5. Group similar modules by performing connected components on the adjacency matrix of step 2.3.4;
2.3.6. Retain the number of clusters formed, $k$, as initial hypothesis
2.3.7. Perform Gaussian mixture model (GMM) clustering by varying the number of GMM components between $\mathrm{min}(1, k-\varepsilon ):k+\varepsilon$. In this study, $\varepsilon =3$ was used
2.3.8. For each GMM, estimate the Bayesian information criterion (BIC)^90^, and retain the solution (number of clusters) with the lowest BIC

It is worth noting that Algorithm 3 can be adjusted for triangular modularization of single-layered shell structures by finding cliques of size three (also called cycles of size three), instead of cliques of size four. Figure 8e presents the results of the tetrahedral modularization. Each colour in Fig. 8e represents the faces of one module (each face of the tetrahedral module includes three cylindrical elements as the edges). In the present study, the SkS was correctly divided into 48 tetrahedral modules with similar edge lengths and a set of connectors between each module.

## Experiments

Four sets of terrestrial laser scanner (TLS) 3D point cloud data using the Leica BLK360 was collected, namely, two datasets from linear free-form SkS (Fig. 1a); one from linear hemispherical SkS (Fig. 1b); and one from cylindrical SkS (Fig. 1c). In this study, four experiments were designed to report on the scientific improvements compared to existing established approaches. Table 1 shows the experiment design, type of analysis and methods of comparison for each of the collected datasets.

**Table 1:** Summary of the experiments presented in the “Results” section.

Experiment	Dataset	Metrics of Validation and Comparison
Linear SkS modeling using Algorithm 2	Free-form SkS (Fig. 1a) Hemispherical dome SkS (Fig. 1b)	Accuracy of final node coordinates to the ground truth (manual/visual nodes)
Bayesian robust neighborhood definition	Free-form SkS (Fig. 1a)	Root mean square error of the detected neighborhood size to the ground truth for the following methods Proposed Bayesian method Particle swarms Particle swarm with Adam gradient^93^ Uniform sampling
SVM-based prominent mode detection	Free-form SkS (Fig. 1a) Hemispherical dome SkS (Fig. 1b)	Object detection accuracy, recall, precision and F-measure for the following methods Proposed SVM clustering k-means clustering Spectral clustering
Generative modeling	Hemispherical SkS dome (Fig. 1b) Cylindrical SkS tower (Fig. 1c);	Presentation of the final results of stochastic generative modeling for Design optimization of geodesic dome Optimal location and number of facilities to build tower

## Results

### Linear SkS modeling using Algorithm 2

**Figure 9:**
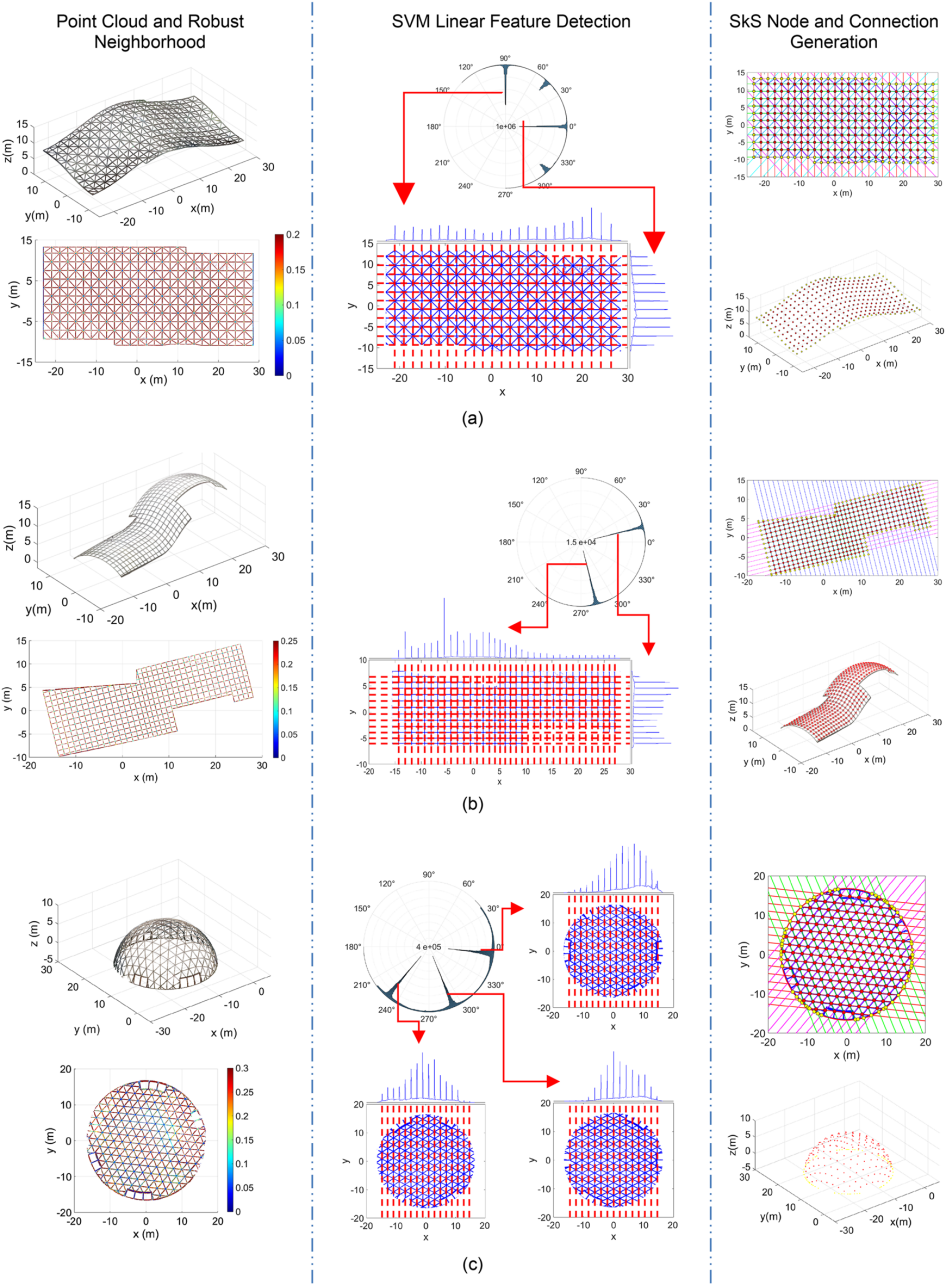
Results of Algorithm 2: (**a**) Free-form SkS with Tetrakis element arrangement; (**b**) Free-form SkS with rectangular element arrangement; and (**c**) Hemispherical geodesic dome with deltille element arrangement.

Figure 9 shows the step-by-step results of applying Algorithm 2 on input SkS point clouds of: (i) the free-form SkS with Tetrakis projected element arrangement (Fig. 9a); (ii) the free-form SkS with rectangular projected element arrangement (Fig. 9b); and (iii) hemispherical geodesic dome with triangular element arrangement (Fig. 9c). The results shown in Fig. 9 include: (i) the input point cloud and the heatmap of the robust neighborhood definition-left; (ii) polar histogram of the directional vector angle from PCA, and the result of the SVM-based linear classification method-middle; and (iii) eligible line intersection and estimation of the locations of nodes in 2D and 3D-right. The mean radial spherical error (MRSE)^94^ is used to report on the accuracy of the estimated node using Algorithm 2 and the ground truth (manual detection). The MRSE for the node estimation was 3.5 mm, 2.8 mm and 3.2 mm for free-form with Tetrakis, free-form with rectangular, and dome with deltille projected member arrangements, respectively. It is worth noting that the point measurement accuracy (together with possible errors in registration) for the BLK360 is reported around 6-8 mm^51^. As such, using the proposed methodology, and by effectively utilizing robust least-squares fitting to minimize the impact of random measurement error, the accuracy of the node estimation was within the range that could only be achieved by more advanced TLS instruments, such as Leica RTC360.

### Bayesian robust neighborhood definition

Table 2 presents the results of the robust neighborhood definition using: (i) particle swarm algorithm (normal and with Adam gradient approximation); (ii) uniform subsampling of neighborhood (with 20 and 40 samples); and (iii) proposed Bayesian optimization (with 10 and 20 evaluations). The results include: (i) the accuracy of the estimated neighborhoods; (ii) the average time for convergence per point; and (iii) the accuracy of the estimated directional vectors’ angles. The results indicate that the Bayesian optimization with 20 evaluations provided the most accurate results for the neighborhood definition, and consequentially directional vector estimation accuracy. Compared to 40 uniformly distributed neighborhood sizes (next best), the accuracy of the estimated directional vector was improved around 50%. The computation time, however, was around 3 times slower than the next best result. Moreover, the results indicated that the PSA achieved the fastest convergence with the least accurate results. The utilization of the numerical gradient correction, inspired by Adam^93^, together with PSA, was, however, found effective.

**Table 2:** Results of the best neighborhood definition optimization.

Method		Neighborhood size accuracy (mm)	Average time for optimization (s)	Accuracy of directional vector estimation (°)
Particle Swarm Algorithm (PSA)	Regular	16.5	**0.05**	0.85
	Adam gradient	11.5	0.09	0.71
Uniform sampling	With 20 samples	12.5	0.08	0.77
	With 40 samples	9.3	0.18	0.62
Proposed Bayesian optimization	With 10 evaluations	10.8	0.31	0.67
	With 20 evaluations	**7.1**	0.62	**0.36**

Significant values are in bold.

### SVM-based prominent mode detection

**Figure 10:**
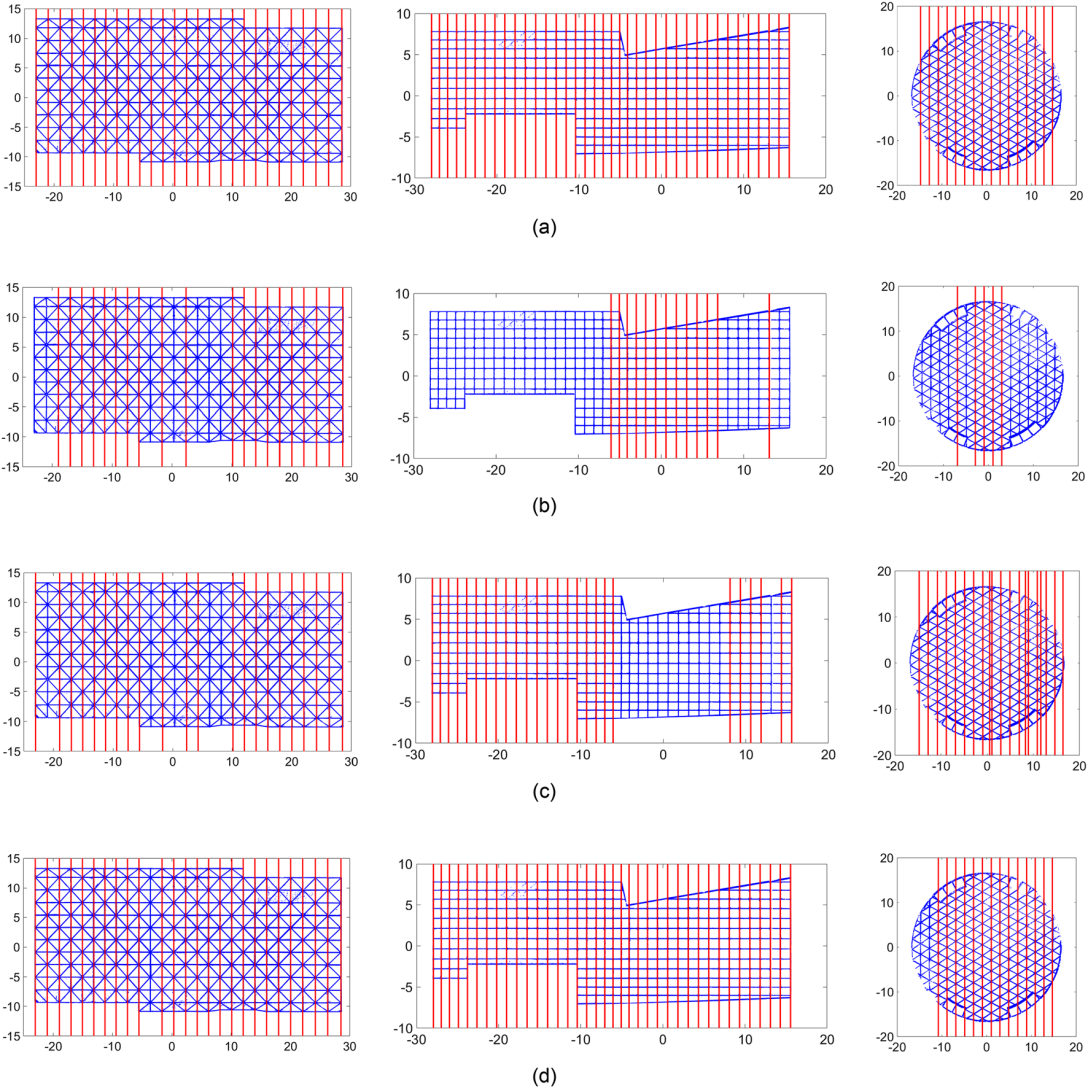
Binary clustering of prominent modes for linear feature detection: (**a**) proposed trained SVM classifier; (**b**) k-means clustering; and spectral clustering with (**c**) 10 neighbours; and (**d**) 20 neighbours.

Figure 10 shows the results of binary clustering of the most prominent modes of the projected rotated points onto the horizontal axis, which represents linear features using: (i) proposed SVM-based decision support, trained using simulated data (Fig. 10a); (ii) k-means clustering (Fig. 10b); and (iii) spectral clustering using 10 and 20 neighbours (Fig. 10c and d). The results show that the k-means clustering as well as spectral clustering with 20 neighbours are prone to Type I errors (not detecting existing linear features), while the spectral clustering using 10 neighbours is prone to both Type I and Type II errors (detecting non-linear features as linear).

Table 3 shows the results of the precision, recall, accuracy, and F-measure for each method. The results demonstrate that the proposed SVM-based method detected all linear features correctly. K-means clustering performed poorly with only 46.91% precision and 64.87% F-measure. Spectral clustering with 20 neighbours performed close to the proposed method, achieving 98.11% F-measure. While spectral clustering shows promise, its performance as a method was impacted by the number of neighbouring data points used to generate the Laplacian matrix, which is not generally known a-priori.

**Table 3:** Results of the linear feature detection using different methods.

Method	Precision (%)	Recall (%)	Accuracy (%)	F-measure (%)
Proposed SVM	**100.00**	**100.00**	**100.00**	**100.00**
k-means	46.91	**100.00**	76.24	63.87
Spectral clustering (10 neighbours)	79.01	92.75	87.85	85.33
Spectral clustering (20 neighbours)	96.30	**100.00**	98.34	98.11

Significant values are in bold.

### Generative modeling

#### Design optimization of geodesic dome

**Figure 11:**
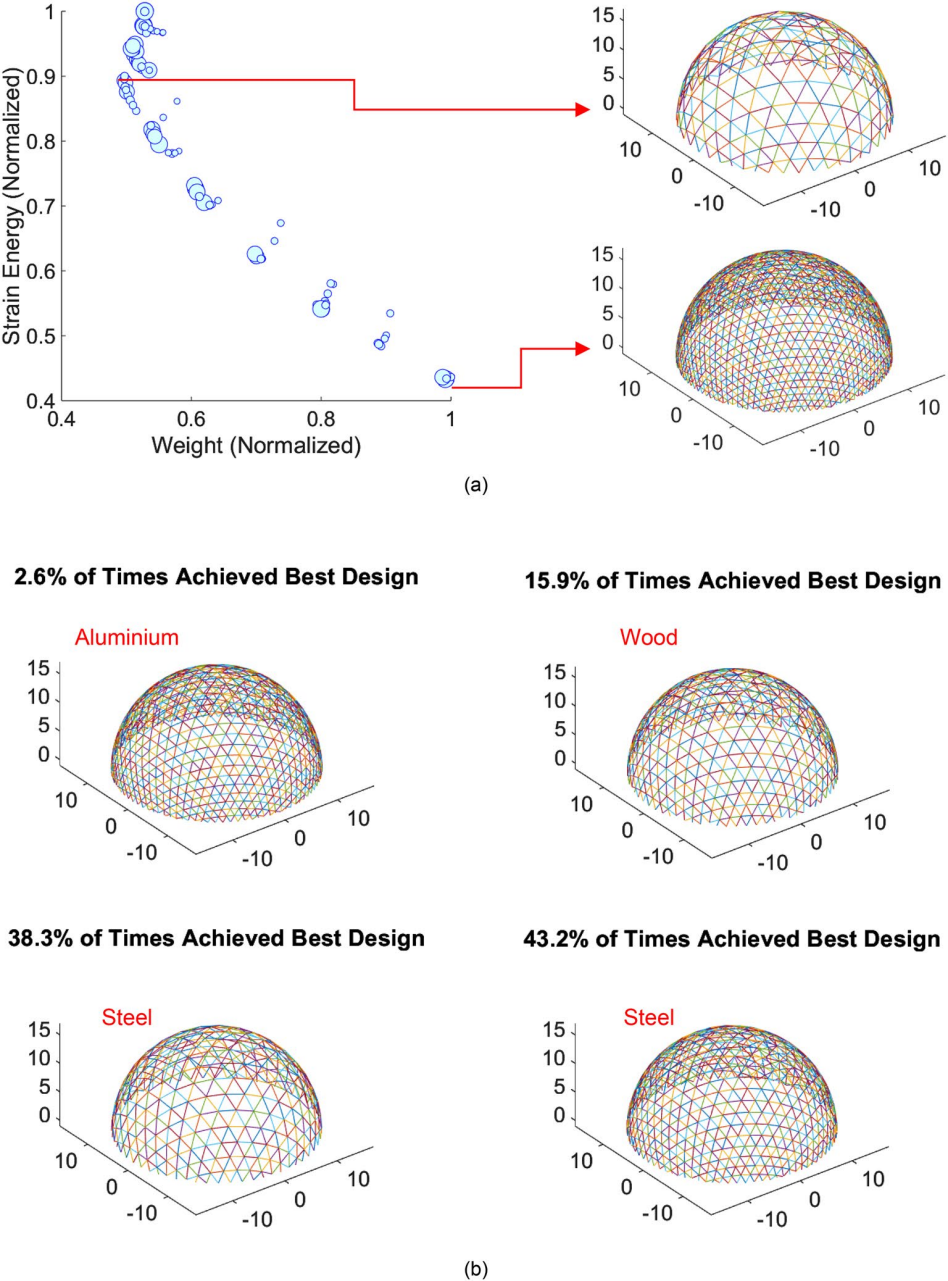
Results of the generative design optimization of geodesic dome: (**a**) sample of Pareto front solutions for steel dome- weight vs. strain energy; and (**b**) results of the stochastic MCDM.

Figure 11 shows the results of the design optimization of the geodesic dome project. Figure 11a-left shows the Pareto front of the normalized weight vs. strain energy for steel geodesic domes (size of points represents the relative cost of dome). Figure 11a-right shows the two designs that achieved minimum weight (top) and minimum strain energy (bottom). As observed, as the strain energy reduces, the weight of the structure increases, and hence, a single global optimum that minimizes both objectives simultaneously cannot be achieved. In fact, the designs that minimize strain energy and weight are vastly different. In this study, other than weight and strain energy, embodied Carbon, embodied energy, volume, construction cost and fundamental frequency requirements must also be achieved. In this study, to report the results of the NDGA II together with the proposed stochastic MCDM, the order of importance of the objective functions (from most to least important) were: Embodied Carbon; Embodied Energy; Construction Cost; Volume; Weight; Fundamental Frequency; and Strain Energy. 1000 sets of seven random weights were generated and assigned to the objective functions based on their ranking. The designs and the percentage of times achieving the least overall objective function value were reported. For each material type, 120 Pareto optimal solutions (total of 360 solutions) were generated. The results of the stochastic MCDM of the 360 Pareto solutions are provided in Fig. 11b.

As illustrated, despite its high strength to weight ratio, the best Aluminum design only achieved best overall design 2.6% of the times, attributed to the highly demanding embodied energy and Carbon of the Aluminum production chain. The best wood design achieved best overall design 15.9% of times. The top two steel designs were the best overall designs 81.5% of the times combined. As such, 360 Pareto front solutions were summarized into four dominant solutions for stakeholder decision support. It is to emphasize that the results are highly dependent on the order of importance of the objectives, and hence, are project specific.

#### Optimal location and number of facilities to build modular tower

**Figure 12:**
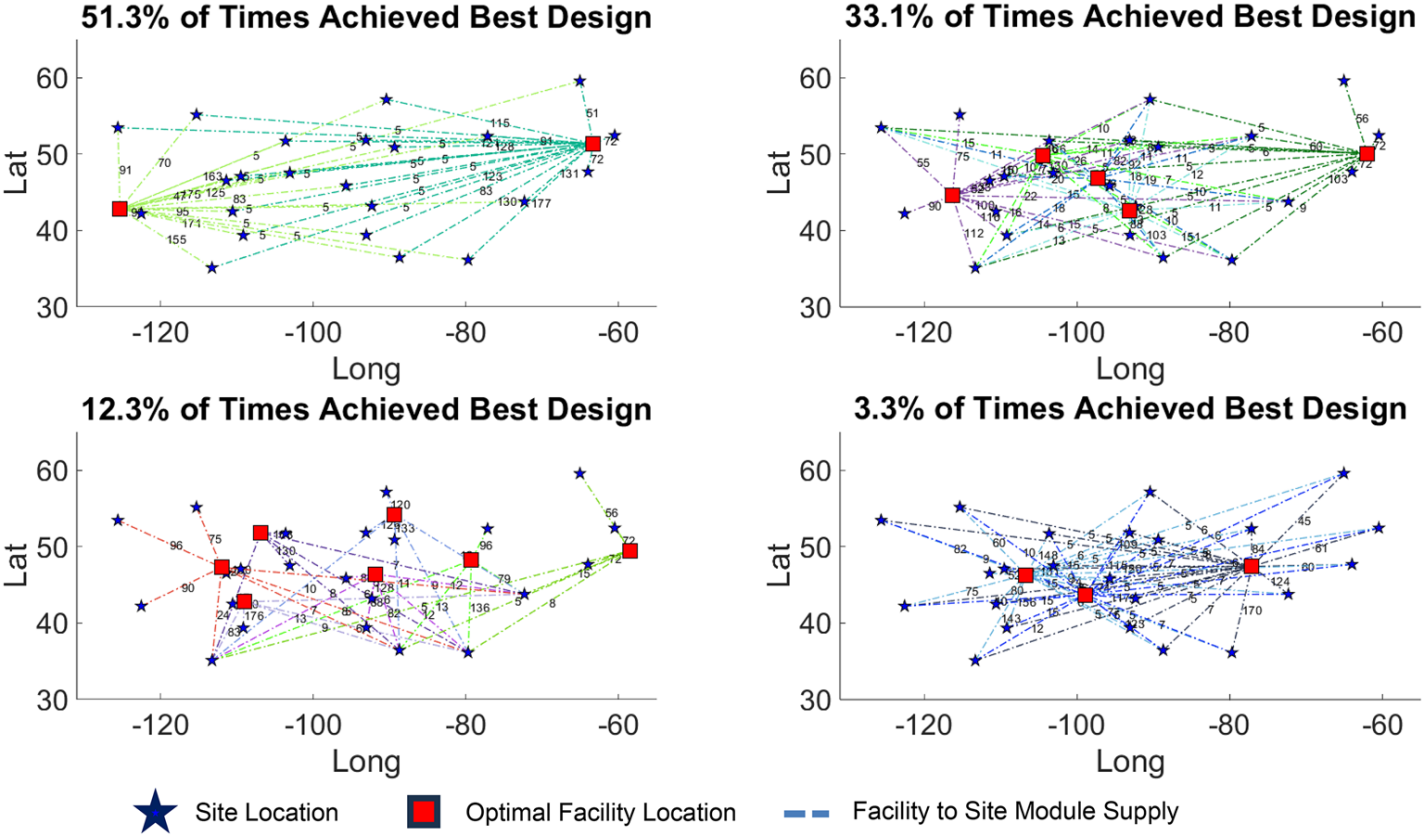
Results of the top four alternatives of the stochastic MCDM-based generative facility location optimization.

The problem of finding the optimal facility number and location to rebuild the SkS tower (Fig. 1c) in different locations across North America (Fig. 8b) under conditions to minimize embodied Carbon and cost of facility, while maintaining high uniformity between facility capacity, was explored. The capacity of the facilities are a function of the module demand for each site. For this, the point cloud acquired from the SkS tower of Fig. 1c was transformed into as-built BIM using Algorithm 2, and further modularized into stable tetrahedral components through Algorithm 3. In this study, the number of facilities are changed from 2 to 7 and for each facility number, 90 Pareto front solutions were generated. Amongst the 540 different solutions, the top four were selected based on the project specific importance criteria. This importance criteria from most important to least important was Embodied Carbon, Cost of Facility, and Uniformity of Capacity. The results of the generative location optimization of the facilities are presented in Fig. 12. The number of modules supplied by each facility to the sites (based on the facility demand) is shown on the dashed connectivity lines. It was observed that the best solution using three, seven, five, and two assembly facilities achieved the best solution 3.3%, 12.3%, 33.1%, and 51.3% of the times, respectively. The results provide a diverse range of options from two to seven facilities for the project team to finalize.

## Discussion

This study presented the first investigation on the application of automated point cloud processing using a newly developed framework, called FIM^®^, for generative redesign and repurposing of existing SkS systems. Three original algorithms were proposed to: (i) generate as-built BIM for two classes of SkS systems, namely, linear and cylindrical; and (ii) modularize SkS designs with repeatable patterns for optimal production, operations and supply chain management in support of mass production and industrialization in construction. The algorithms proposed several AI-based scientific contributions to existing literature. These included: (i) supervised SVM-based binary decision support, trained using simulated data, for rule-of-thumb definition of linear features, prominent mode detection, and elliptic pattern detection; (ii) Bayesian optimization for selecting the neighborhood achieving MCD (the outlier-free set); (iii) unsupervised GMM-based Bayesian clustering of similar tetrahedral modules; and (iv) Monte Carlo-based stochastic MCDM for selection of top four generative optimization solutions. The application of these methods was investigated on four real-world projects to solve two separate generative modeling problems, namely, design optimization, and assembly facility location optimization. The four sets of point cloud data were acquired from the four projects using the Leica BLK360. To help generate optimal solutions, the NSGA II algorithm was utilized to provide many Pareto front solutions.

The results of the experiments indicated that Algorithm 2 achieved SkS node estimation accuracy between 2.8 and 3.5 mm, improving the single point measurement accuracy of the host laser scanner, the BLK360, by 50–70%. The proposed Bayesian optimization improved the accuracy of the directional vector estimation of point neighborhoods using PSA and uniform sampling by 34% and 27% on average. The proposed SVM-based linear feature detection outperformed line detection using k-means, and spectral clustering by an average of 56% and 9%, respectively. The proposed modularization of the SkS tower correctly identified all modules of the best set of tetrahedral modules. Finally, two separate generative optimization problems were revisited. It was observed that the NSGA II algorithm together with the stochastic MCDM can be utilized to provide unique and diverse best four solutions as a function of project-specific importance criteria. This demonstrates promise for future use of the framework to create many training datasets for generative adversarial networks that can generate new and original designs using only stakeholder-defined criteria.

While the results of the study show promise for the proposed framework, the following avenues for future development are recommended:

- Extension of the current system for other types of SkS such as triple-layered grids and elements with rectangular (and rounded rectangular) cross-sections.
- Utilization of the best designs and their performances to train a generative adversarial network to generate completely new designs given only the set of stakeholder requirements and constraints.
- Extension of the modularization framework for other types of BIM-based objects, such as walls, floor, columns, and beams in typical residential buildings.
- Evaluation of the effectiveness of other multi-objective evolutionary optimization algorithms, including decomposition-based, such as MEOA/D^95^ and its variants^96^ and newer dominance-based for many-objective optimization, such as unified NSGA III^97,98^, to generate the Pareto Front solutions faster and with higher diversity.

## Data Availability

The datasets used and/or analysed during the current study available from the corresponding author on reasonable request.
